# Safety and complications of upper pole puncture in supine percutaneous nephrolithotomy

**DOI:** 10.1007/s00345-026-06735-0

**Published:** 2026-10-14

**Authors:** Juliana Villanueva-Congote, Yi W. Li, Michal Segall, Mehmet Gokce, Silvia Proietti, Guido Giusti, Jose Ernesto Torres Garcia, Braulio Manzo, Alan Yaghoubian, Mariano Gonzalez, Leye Ajayi, Daniel A. Wollin, Brian H. Eisner

**Affiliations:** 1 Department of Urology, Massachusetts General Hospital, Harvard Medical School, Boston, USA; 2 Department of Urology, Georgetown University, Washington, DC USA; 3 Department of Urology, Ankara University School of Medicine, Ankara, Turkey; 4 Department of Urology, San Raffaele Hospital, Milan, Italy; 5 Department of Urology, Hospital Angeles Leon, Guanajuato, Mexico; 6 Department of Urology, University of California in Los Angeles, Los Angeles, USA; 7 Department of Urology, Hospital Italiano de Buenos Aires, Buenos Aires, Argentina; 8 Department of Urology, Royal Free Hospital, London, England; 9 Department of Urology, Brigham and Women’s Hospital, Boston, USA; 10 Department of Urology, Tulane University, 4224 Houma Blvd, Suite 640 Metairie, New Orleans, LA 70006 USA

**Keywords:** Supine, Percutaneous nephrolithotomy, Renal calyx

## Abstract

**Objective:**

Supine percutaneous nephrolithotomy (PCNL) has gained attention as a viable alternative to the traditional/historical prone approach for the treatment of large renal stones.

However, concerns remain regarding the safety and feasibility of upper calyx punctures in the supine position, particularly for upper-pole access. This multicenter, retrospective cohort study evaluated the safety and stone-free rates of upper-pole punctures in supine PCNL, comparing outcomes with middle and lower calyx punctures and with literature on prone PCNL.

**Methods:**

This retrospective study analyzed supine PCNL cases performed between 2009 and 2019 across eight international centers, categorizing patients by calyx of entry (upper, middle, or lower). Patients who required multiple calyx entries were excluded. Demographics, surgical details, and outcomes were recorded, including complications and stone-free rates. We used logistic regression to assess the association between stone-free rates and calyx puncture sites, adjusting for age, gender, Guy’s Stone Score, and ASA score. Based on the initial findings, we performed a second multivariable logistic regression among patients undergoing upper calyx puncture using the same covariates. ANOVA was used to compare the means of quantitative variables, and Chi-square tests assessed associations between categorical variables. Bonferroni adjustment was applied when appropriate.

**Results:**

Among 989 patients, 10% had upper, 28.5% middle, and 61.5% lower calyx punctures. No significant differences were found in overall complication rates when comparing upper, middle, and lower calyces. Thoracic complications were rare and did not differ significantly across calyces (*p* = 0.74). Upper calyx punctures had lower stone-free rates (51%) compared to middle (77.7%) and lower (62.8%) punctures (*p* < 0.001). The overall complication rate did not differ significantly (*p* = 0.82). According to the logistic regression, patients with a middle puncture site had 2.98 times higher odds of a stone-free outcome than those with an upper puncture site (OR, 2.98; 95% CI, 1.69–5.29). In contrast, patients with a lower puncture site did not have a significant difference in stone-free rates compared to those with an upper puncture (OR = 1.10, 95% CI: 0.64, 1.86, *p* = 0.7). Among patients undergoing upper calyx puncture, Guy’s Stone Score 4 was independently associated with lower odds of achieving stone-free status (OR 0.20, 95% CI 0.04–0.91; *p* = 0.045). No significant associations were observed for age, gender, or ASA classification.

**Conclusion:**

Upper calyx punctures showed similar complication rates to middle and lower calyx punctures, with slightly lower stone-free rates likely due to higher stone complexity. These findings support the feasibility, safety, and efficacy of supine PCNL for upper calyx access.

## Introduction

Supine percutaneous nephrolithotomy (PCNL) has emerged as a promising alternative to the traditional prone approach to large renal stones [1]. Studies have suggested that upper calyceal access during supine PCNL — whether supracostal or subcostal — achieves comparable stone-free rates to prone PCNL while reducing operative time [2]. However, some critics of the supine position have suggested that upper calyx puncture is more challenging in the supine position and carries an unacceptable complication risk, including risk of thoracic and bleeding complications and unacceptable stone-free rates [3]. Furthermore, while supine PCNL may be appropriate for lower calyceal stones, some have argued that the prone position is preferable for upper pole punctures, claiming improved outcomes with prone PCNL in such cases [4]. However, there is limited evidence to support this claim.

We aim to address these concerns through a multi-center, multinational, retrospective cohort review of supine PCNL procedures. Our objectives are twofold. First, to compare the safety and the stone-free rates of upper-pole supine PCNL with those targeting the middle and lower calyces, and second, to compare the safety and stone-free rates of our upper pole punctures in the supine position to the historical literature of prone upper pole puncture for PCNL.

We hypothesize that upper calyceal punctures in the supine position offer a safe and feasible access route, with complications and stone-free rates comparable to middle and lower calyceal punctures, as well as upper calyceal punctures in the prone position.

## Methods

Following all centers’ approval from the Institutional Review Board, we reviewed the electronic medical records of consecutive surgical patients who underwent supine PCNL across eight institutions in Argentina, England, Italy, Mexico, Turkey, and the United States from 2009 to 2019. Patients were grouped based on the calyx of entry during surgery. Patients who underwent multi-calyx puncture were excluded from the study.

PCNL was performed in the supine position according to each institution’s standard surgical technique. No standardized respiratory maneuver was employed during renal access. The use of breath-holding, the Valsalva maneuver, or no respiratory maneuver was left to the discretion of the operating surgeon.

Baseline characteristics, including age, gender, body mass index (BMI), and medical histories, including diabetes and urological conditions, were recorded.

The primary outcome of interest was the occurrence of postoperative complications. Complications were categorized using the Clavien-Dindo classification system. For patients with multiple Clavien-Dindo complications, only the highest grade was recorded.

Secondary outcomes included postoperative metrics such as length of hospital stay, total operative time, and stone-free rate. Stone-free rate was assessed as a dichotomized variable, considering how many patients were completely free of kidney stones after treatment. Those without imaging follow-up were excluded from the analysis. Stone-free rates were determined based on follow-up imaging, which included CT, KUB, or ultrasound, as per the discretion of each site. The criteria for determining stone-free status were site-specific.

Guy’s Stone Scores were categorized into complex and non-complex stone groups. Complex stones were defined as a score of ≥ 2, while non-complex stones corresponded to a score of 1.

### Statistical analysis

Continuous variables were assessed for normality using the Shapiro-Wilk test. Normally distributed variables are presented as mean ± standard deviation and were compared using one-way analysis of variance (ANOVA), whereas non-normally distributed variables are presented as median (interquartile range [IQR]) and were compared using the Kruskal-Wallis test. Categorical variables are presented as frequencies and percentages and were compared using Pearson’s Chi-square test or Fisher’s exact test, as appropriate.

Initially, we used a multivariate logistic regression model to assess the association between stone-free rates and calyx puncture sites. Covariate selection followed a clinical and literature-based approach, including age, gender, Guy’s Stone Score, and ASA score. Based on the findings of the initial model, a second multivariable logistic regression analysis was performed to identify factors independently associated with stone-free status among patients undergoing upper calyx puncture. Because of the small number of patients in each ASA category, ASA scores were grouped as 1 and 2 versus 3 and 4. The same covariates as in the initial model (age, gender, Guy’s Stone Score, and ASA classification) were retained.

A significance threshold of *p* ≤ 0.05 was used. However, a Bonferroni adjustment was applied to account for multiple comparisons when conducting multiple chi-square tests. Data analysis was done using R Studio.

## Results

### Demographics and patient characteristics

Between 2009 and 2019, 1,022 patients who underwent supine PCNL were entered into our database. Thirty were excluded due to lack of follow-up, and three due to missing operative time data, leaving a final cohort of 989 cases. Of these, 10% (99/989) underwent upper caliceal puncture, 28.5% underwent middle caliceal puncture, and 61.5% underwent lower caliceal puncture. Among patients undergoing upper calyx puncture, all procedures (100%) were performed via a supracostal approach. There was no statistically significant difference between the groups regarding patient age and gender. Among patients undergoing lower calyx punctures, 46.4% were white, compared to 70.9% for middle calyx punctures and 42.4% for upper calyx punctures (*p* < 0.001). ASA I was most prevalent in the middle (53.9%) and lower (42.9%) calyx punctures, whereas ASA II and III were more commonly observed in the upper calyx group (*p* < 0.01). See Table 1**and** Table 2 for demographic and patient characteristics.

**Table 1:** Demographics

Characteristics	Upper Calyx Puncture (*N* = 99)	Middle Calyx Puncture (*N* = 282)	Lower Calyx Puncture (*N* = 608)	*p*-value
Age (SD)	52.8 (13.5)	51.9 (15.1)	50.6 (15.1)	0.251
Gender N (%)				0.15
Male	55 (55.6)	110 (39.0)	256 (42.1)	
Female	44 (44.4)	172 (61.0)	352 (57.9)	
Laterality N (%)				0.037**
Right Side	50 (50.5)	108 (38.3)	245 (40.3)	
Left Side	43 (43.4)	137 (48.6)	313 (51.5)	
Bilateral	6 (6.1)	37 (13.1)	50 (8.2)	
Ethnicity N (%)				< 0.001*
White	42 (42.4)	200 (70.9)	282 (46.4)	
Hispanic	27 (27.3)	58 (20.6)	271 (44.6)	
Not reported/Other	30 (30.3)	24 (8.5)	55 (9.0)	
ASA Score N (%)				< 0.001*
ASA I	29 (29.3)	152 (53.9)	261 (42.9)	
ASA II	43 (43.4)	76 (27.0)	218 (35.9)	
ASA III	19 (19.2)	36 (12.8)	65 (10.7)	
Guy’s Stone Score N (%)				
Score 1	16 (16.2)	77 (27.3)	197 (32.5)	0.003 *
Score 2, 3, or 4	67 (67.7)	177 (62.8)	324 (54.0)	0.0027*
Not specified	16 (16.2)	28 (9.9)	87 (13.5)	0.031**

*Significant after Bonferroni Adjustment

**Not significant after Bonferroni Adjustment

**Table 2:** Medical History

Characteristics	Upper Calyx Puncture		Middle Calyx Puncture		Lower Calyx Puncture	
N (%)	99	(%)	282	(%)	608	(%)
Diabetes (%)	23	23.23	50	17.73	73	12.01
Multiple Sclerosis (%)	1	1.01	4	1.42	5	0.82
Anticoagulation (%)	2	2.02	7	2.48	12	1.97
Antiplatelet Medication (%)	14	14.14	32	11.35	54	8.88
History of recurrent UTI (%)	17	17.17	61	21.63	110	18.09
History of Recurrent Pyelonephritis (%)	6	6.06	24	8.51	50	8.22
History of Urosepsis (%)	2	2.02	13	4.61	16	2.63
Previous PCNL (%)	10	10.10	49	17.38	98	16.12

Upper calyx access was more frequently linked to higher stone complexity when assessing stone complexity by puncture site. Specifically, upper calyx punctures were associated with the highest proportion of complex stones (Guy’s Scores 2, 3, and 4) and the lowest proportion of simple stones (Guy’s Score 1) treated. Only 16% of upper calyx stones were Guy’s Stone Score 1 compared with 27.3% for the middle calyx and 32.5% for the lower calyx (*p* = 0.003). (Table 1)

### Postoperative outcomes

Overall, there were six thoracic complications: one associated with upper pole access, one with middle calyx access, and four with lower pole access (*p* = 0.74). Additionally, there were 38 non-thoracic complications (*p* = 0.95). The overall complication rates did not differ significantly between the groups (*p* = 0.82). For a detailed summary of complications, see Table 3.

**Table 3:** Clavien-Dindo Complication Classification

Complication Type	Upper Calyx Puncture	Middle Calyx Puncture	Lower Calyx Puncture	Clavien-Dindo Classification
Total, N (%)	99	282	608	
None	94 (94.9)	270 (95.7%)	575 (94.6%)	-
Non-thoracic Complications				
Hematuria	1 (0.01)	1 (0.004)	2 (0.003)	I
Renal Hematoma	0	0	1(0.002)	I
Pain	2 (0.02)	1 (0.004)	0	II
Transfusion	0	1 (0.4%)	1 (0.2%)	II
Fever	0	1 (0.004)	3 (0.05)	II
Pyelonephritis	0	0	1(0.002)	II
UTI	0	0	1(0.002)	II
Bleeding	0	2 (0.007)	3 (0.05)	II
Embolization	1 (0.01)	4 (0.014)	6 (0.01)	III
Succinylcholine toxicity	0	0	1(0.002)	IV
ICU Admission	0	1 (0.004)	2 (0.003)	IV
Vasopressor Requirement	0	0	2 (0.003)	IV
Thoracic Complications				
Thoracic Complications	1 (0.01)	1 (0.004)	4 (0.007)	III
Not Specified Complications				
Unspecified	0	1 (0.64)	6 (0.01)	-

There were no statistically significant differences in overall (*p* = 0.82), thoracic (*p* = 0.74), or non-thoracic (*p* = 0.95) complication rates among the puncture groups

Additionally, we found a significant difference in stone-free rates based on calyx of entry, where upper caliceal puncture had a lower stone-free rate at 51% compared to 77.66% and 62.83% for middle and lower calyceal puncture, respectively. For the subset of cases where operative time was available, lower calyx puncture had the longest operative time (105.8 min ± 51.02, *p* < 0.001). See Table 4.

**Table 4:** Postoperative Outcomes * Definition of stone-free status was institution-dependent

Characteristics	Upper Calyx Puncture		Middle Calyx Puncture		Lower Calyx Puncture		*p*-value
N (%)	99	(%)	282	(%)	608	(%)	
Stone Free Rate (%)*	51	51.52	219	77.66	382	62.83	< 0.001
Length of stay in days (SD)	1.01 (1.36)		0.52 (1.14)		1 (1.56)		< 0.001
Operative Time (SD)	90.842 (34.84)		94.426 (40.47)		105.84112 (51.02)		< 0.001

**Table 5:** Association Between Puncture Site and Stone-Free Rates

Variables	Univariable Analysis			Multivariable Analysis		
Puncture Site	Odds Ratio	95% CI	P value	Odds Ratio	95% CI	p-value
Upper Calyx	Reference Category			Reference Category		
Middle Calyx	3.75	2.23, 6.31	< 0.001	2.98	1.69, 5.29	< 0.001
Lower Calyx	1.53	0.98, 2.38	0.062	1.10	0.74, 1.97	0.4

Model adjusted by age, gender, Guy’s Stone Score, and ASA score

According to the logistic regression, patients with a middle puncture site had 2.98 higher odds of stone-free outcome than those with an upper puncture site (OR = 3.07, 95% CI: 1.67, 5.65, *p* < 0.001). In contrast, patients with a lower puncture site did not have a significant difference in stone-free rates compared to those with an upper puncture (OR = 1.10, 95% CI: 0.64, 1.86, *p* = 0.7)—Table 5.

The second multivariable logistic regression model showed that, among patients undergoing upper calyx puncture, only Guy’s Stone Score 4 was independently associated with lower odds of achieving stone-free status compared with Guy’s Stone Score 1 (OR: 0.20, 95% CI: 0.04–0.91; *p* = 0.045). No significant associations were observed for age, gender, or ASA classification.

## Discussion

When analyzing the supine approach for PCNL procedures, two key considerations arise. First is the feasibility of the supine approach for all stone locations, particularly whether there are different outcomes for stones located in the upper, middle, or lower calyces. Second, is an evaluation of the risks associated with the supine versus prone approach, especially for stones in the upper calyx, which are often cited as having a higher risk of thoracic puncture.

### Comparison of calyces

The stone-free rates across different calyx locations revealed a significant difference (*p* < 0.001) between access sites. The highest stone-free rate was seen in the middle calyx puncture. These findings align with one study by Gokce et al., which reported that the middle calyx provided the best access to all renal segments, partially explaining the higher stone-free rates observed in our study [5]. The lower calyx puncture followed in stone-free rates, while the upper calyx puncture had the lowest. These findings differ from the results of Singh et al., who also evaluated the efficacy of upper calyceal versus lower calyceal puncture and suggested that the upper calyx approach provided similar clearance rates [6]. Nevertheless, in our study, the highest proportion of complex stones was observed in cases managed through upper calyx punctures, which had the greatest percentage of Guy’s Stone Score 2, 3, and 4 stones and the lowest percentage of Guy’s Stone Score 1 stones. We surmise that this uneven distribution of more complex stones was the main driver of the lower stone-free rates observed in the upper calyx access group, as our logistic regression showed that the upper calyx access alone was not associated with lower stone-free rate and that the effect is likely driven primarily by the underlying complexity of the stone.

Regarding postoperative complications, we found no statistically significant differences when comparing upper, middle, and lower calyces. However, the length of stay and postoperative time were significantly longer in the lower calyx puncture. Nevertheless, we believe these statistically significant differences were not clinically meaningful.

### Supine vs. prone for upper calyx

Numerous large meta-analyses have highlighted the advantages of supine PCNL over prone PCNL, citing benefits such as improved patient positioning and enhanced safety, lower transfusions and fever rates, and [7] shorter anesthesia time [8]. Yet, the majority of PCNL cases worldwide continue to be performed in the prone position [9] as critics still argue that supine PCNL procedures carry unacceptable risks, including thoracic complications during upper pole punctures, historically reported to reach rates as high as 15% [10]. Additionally, some suggest that the stone-free rates generally remain greater in prone position [11] and that the upper pole access is more easily accessible in the prone position [12].

Our findings reveal a complication rate below 10%, with hemorrhage requiring embolization being the most prevalent major complication, occurring in 11 patients (1.1%).

Specifically, we observed six thoracic complications and 38 non-thoracic complications. Importantly, no statistically significant differences were identified between thoracic complication rates (*p* = 0.74) and overall complication rates (*p* = 0.82) when comparing upper, middle, and lower calyces in our study. These results suggest that upper pole access is feasible and safe in the supine position.

Wood et al. compared supine and prone upper pole punctures, suggesting that upper pole access in supine PCNL surgeries is equally safe compared to prone PCNL surgeries [3]. Their study reported no significant differences in complication rates and had similar operative time and blood transfusion rates between groups [3]. However, other articles debate which position offers improved operative time. While some have concluded that prone PCNL leads to reduced operative time [7], others report the opposite [13], indicating that operative time may not be as pivotal in differentiating the two procedures as previously emphasized.

Additionally, He et al. concluded in their meta-analysis that supracostal access, which is typically upper pole access, was adequate but less safe than infracostal access in PCNL due to reduced hemoglobin levels and an increased risk of hydrothorax [14]. However, their analysis was restricted to prone-positioned patients, unlike our study. In our cohort, none of the patients undergoing upper calyx puncture required transfusion, and complication rates did not vary significantly between groups. We also found no differences in ICU admissions (*p* = 0.84). Notably, our multi-institutional cohort of 989 patients is comparable in scale to the meta-analysis conducted by He et al., offering robust data to support our findings while also highlighting intriguing insights into the feasibility and safety of upper calyx access in the supine position.

One of the key strengths of our study is its analysis of supine PCNL outcomes in a large, diverse, international cohort, enhancing the generalizability of our findings. This study provides practical insights into supine PCNL and its applicability by capturing data from multiple institutions and practice settings. Nevertheless, several limitations should be acknowledged. As a retrospective study, unmeasured variables may have influenced the choice of surgical access, potentially affecting the results. Additionally, tract caliber (standard vs. mini-PCNL) and the use of flexible nephroscopy were not consistently captured across participating centers. As these clinically relevant variables may influence both bleeding and stone-free outcomes, their absence precluded adjustment for potential confounders, and this should be considered when interpreting our findings. Moreover, information regarding the imaging modality used for renal access was not consistently available across participating centers and therefore could not be included in the analysis.

Furthermore, while we compare our findings with the existing literature, the lack of a cohort undergoing prone PCNL at the same institutions limits direct comparisons. Given the multicenter design, follow-up imaging protocols varied between sites and surgeons; therefore, the definition of stone-free status was institution dependent. Upper pole access was selected based on patient anatomy, stone burden, or surgeon preference, introducing potential selection bias. Finally, procedures were performed by specialized surgeons with extensive experience in supine PCNL, which may limit the generalizability of our findings to broader urological practice.

To address the limitations inherent to retrospective analyses, future prospective or randomized studies, along with the evaluation of tools - such as intraoperative ultrasound - to reduce the risks of thoracic complications of upper pole puncture, are needed.

## Conclusion

In this large multinational study, upper calyx punctures in supine PCNL demonstrated comparable safety profiles to middle and lower calyx punctures, with no increase in complications, transfusions, or vasopressor requirements. Although stone-free rates were slightly lower for upper punctures, the higher complexity of stones likely influenced these results. Our findings suggest the feasibility of upper calyx punctures in supine PCNL, offering reassurance to surgeons considering this approach.

## Data Availability

Data is available upon reasonable request to the corresponding author.

## References

[1] Wang H, Yang Z, Chang X, Wang Y, Li J, Han Z (2023) Oblique supine position versus prone position for percutaneous nephrolithotomy: a systematic review and meta-analysis. Videosurgery Other Miniinvasive Techniques 18(2):244–253 10.5114/wiitm.2023.126453 PMC10481440 37680735

[2] Sukumar S, Nair B, Kumar PG, Sanjeevan KV, Bhat HS (2008) Supracostal access for percutaneous nephrolithotomy: less morbid, more effective. Int Urol Nephrol 40(2):263–267 10.1007/s11255-007-9270-2 17899435

[3] Wood GJA, Torricelli FCM, Vicentini FC, Srougi M, Mazzucchi E (2017) Supracostal punctures in supine percutaneous nephrolithotomy are safe. Can J Urol 24(2):8749–8758 28436363

[4] Vicentini F, Gomes C, Danilovic A, Chedid Neto E, Mazzucchi E, Srougi M (2009) Percutaneous nephrolithotomy: Current concepts. Indian J Urol 25(1):4 10.4103/0970-1591.44281 PMC2684301 19468422

[5] Gokce MI, Mazzon G, Nguyen D, Perez BM, Ibis MA, Zeng G et al Navigating Entire Collecting System During Supine Percutaneous Nephrolithotomy: Is Rigid Nephroscopy Enough? A Prospective Study by International Alliance of Urolithiasis Supine Percutaneous Nephrolithotomy Working Group. J Laparoendosc Adv Surg Tech A [Internet]. 2024 May 24 [cited 2025 Apr 2];34(5):415–9. Available from: https://pubmed.ncbi.nlm.nih.gov/38364181/10.1089/lap.2024.0025 38364181

[6] Singh R, Kankalia S, Sabale V, Satav V, Mane D, Mulay A et al (2015) Comparative evaluation of upper versus lower calyceal approach in percutaneous nephrolithotomy for managing complex renal calculi. Urol Ann 7(1):31 10.4103/0974-7796.148591 PMC4310113 25657540

[7] Valdivia JG, Scarpa RM, Duvdevani M, Gross AJ, Nadler RB, Nutahara K et al (2011) Supine Versus Prone Position During Percutaneous Nephrolithotomy: A Report from the Clinical Research Office of the Endourological Society Percutaneous Nephrolithotomy Global Study. J Endourol 25(10):1619–1625 10.1089/end.2011.0110 21877911

[8] Awan AS, Khalid S, Khan SA, Mithani S, Shaikh J, Sharif I (2019) Supine PCNL is the Way Forward, with Reduced Anesthesia and Operative Times As Compared to Prone PCNL, Along with Comparable Blood Loss and Stone Free Rates. J Urol Surg 6(1):1–6

[9] Kamphuis GM, Baard J, Westendarp M, de la Rosette JJMCH (2015) Lessons learned from the CROES percutaneous nephrolithotomy global study. World J Urol 33(2):223–233 10.1007/s00345-014-1367-5 25100624

[10] Lojanapiwat B, Prasopsuk S (2006) Upper-Pole Access for Percutaneous Nephrolithotomy: Comparison of Supracostal and Infracostal Approaches. J Endourol 20(7):491–494 10.1089/end.2006.20.491 16859462

[11] Birowo P, Tendi W, Widyahening IS, Rasyid N, Atmoko W (2020) Supine versus prone position in percutaneous nephrolithotomy: a systematic review and meta-analysis. F1000Res 9:231 10.12688/f1000research.22940.1 PMC7509599 33014345

[12] Carrion DM, Cansino JR, Quintana LM, Gómez Riva J, Mainez Rodriguez JA, Pérez-Carral JR et al (2018) Prone percutaneous nephrolithotomy: its advantages and our technique for puncture. Transl Androl Urol 7(6):950–959 10.21037/tau.2018.10.04 PMC6256044 30505733

[13] Maiti K, Khare KD, Pal DK (2024) Supine versus prone percutaneous nephrolithotomy in management of patient with complex renal stone diseases. Urol J 91(3):558–562 10.1177/03915603241229801 38497204

[14] He Z, Tang F, Lu Z, He Y, Wei G, Zhong F et al Comparison of Supracostal and Infracostal Access For Percutaneous Nephrolithotomy: A Systematic Review and Meta-Analysis. 16 10.22037/uj.v0i0.4727 30882159

